# Rice Bran Fermented with Kimchi-Derived Lactic Acid Bacteria Prevents Metabolic Complications in Mice on a High-Fat and -Cholesterol Diet

**DOI:** 10.3390/foods10071501

**Published:** 2021-06-28

**Authors:** Sihoon Park, Hae-Choon Chang, Jae-Joon Lee

**Affiliations:** Department of Food and Nutrition, Chosun University, Gwangju 61452, Korea; sihun6312@naver.com (S.P.); hcchang@chosun.ac.kr (H.-C.C.)

**Keywords:** fermented rice bran, kimchi-derived lactic acid bacteria, high-fat, high-cholesterol, anti-obesity, anti-dyslipidemia

## Abstract

This aim of this study was to investigate the potential beneficial effects of rice bran powder, fermented by *Weissella koreensis* DB1 isolated from kimchi, to protect against obesity and dyslipidemia induced by a high-fat and high-cholesterol diet, in a mouse model. Male mice were fed a modified AIN-93M diet containing high fat/high-cholesterol (HFCD), or same diet supplemented with non-fermented rice bran powder (HFCD-RB) or fermented rice bran powder (HFCD-FRB) for 10 weeks. In the HFCD-FRB group, body weight, liver and white fat pads weights, triglyceride (TG), total cholesterol (TC), non-high-density lipopreotein cholesterol (non-HDL-C), insulin, glucose and leptine levels in serum, TG levels and the ratio of fat droplets in the liver, TG levels and fat cell size in adipose tissue were decreased, and (high-density lipopreotein cholesterol) HDL-C and adiponectin levels in serum were increased, compared with the HFCD group. The HFCD-FRB group had significantly lower CCAAT-enhancer-binding potein α (C/EBPα), sterol regulatory element-binding transcription protein-1c (SREBP-1c), fatty acid synthase (FAS), and acetyl CoA carboxylase (ACC) gene expression when compared to the HFCD group. The anti-obesity and hypolipidemic effects were marginally greater in the HFCD-FRB group than in the HFCD-RB group. These results suggest that fermented rice bran powder by *Weissella koreensis* DB1 may have potential beneficial effects on the obesity-related abnormalities and the dysfunction of lipid metabolism.

## 1. Introduction

Rice bran (RB) refers to compounds such as pericarp, seed coat, and aleurone layer in the coating of rice, removed through the process of milling brown rice to white rice, and accounts for more than 10% of rice weight. Despite its nutritional value, RB is mostly used as feed for livestock or discarded as agricultural waste, with only a small portion being used to produce RB oil because it undermines rice palatability and hinders storage. RB consists of 12–16% protein, 20–25% dietary fiber, and 16–99% lipid [1], and with recent findings indicating that it also contains various useful ingredients such as phenolic compounds, flavonoide, γ-oryzanol, γ-aminobutyric acid, phytic acid, ferulic acid, arabinoxylan, arabinogalactan, β-sitosterol, tocotrienols, and β-glucan, it has been identified as a new functional ingredient [2,3,4,5]. The phenolic compounds of RB improve lipid metabolism, prevent fatty liver [6], and protect the liver [7], while RB proteins improve blood lipid metabolism and have antitumor effects [8]. RB fibers improve intestine functions [9] and reduce blood glucose levels [10,11], and RB oil has anti-oxidant effects, suppresses the elevation of blood pressure [12], and relieves chronic inflammation [13]. In addition, γ-oryzanol and ferulic acid in RB improve lipid metabolism [14,15] and have antioxidant effects [5,16,17,18]. Thus, there is growing interest in RB as a potential functional food ingredient amid new research findings on various bioactive substances in RB and their effects.

RB consists of hemicellulose cell walls that cannot be digested by humans, which hinders the full utilization of its benefits. Thus, developing functional food materials using RB involves the degradation of carbohydrates, such as enzyme breakdown, fermentation, and compression, in order to break down hemicellulose, which enhances the rate of absorption of functional ingredients in RB. Further, while polyphenols in the food also have a low absorption rate and bioavailability [19,20], fermentation by lactic acid bacteria (LAB) increases their content and bioactivity [5,21,22,23] and converts them into a form that can be absorbed by the intestines, thereby increasing their absorption rate and bioavailability [19,24]. In other words, fermenting natural food materials with LAB imparts various flavors and textures to the food, enhances the value of the food by producing nutritious and functional ingredients, and improves the storage stability of the food through the biosynthesis of antimicrobial substances [25].

Kimchi is a classic LAB-fermented food of the Republic of Korea, and various microorganisms are known to be involved in its fermentation process. The primary types of LAB involved in kimchi fermentation are genus *Lactobacillus* and *Leuconostoc* [26], and these are the major LAB involved in food fermentation. With recent advances in microbial identification techniques, LAB in the genus *Weissella* have been newly isolated and identified [27]. In particular, several studies have been conducted on the isolation, identification, and properties of ornithine-producing *Weissella* strains isolated from kimchi [28,29,30]. L-ornithine is widely used in the United States and other countries as a food material that enhances muscle synthesis by increasing the secretion of growth hormones and prevents obesity by facilitating basal metabolism [31,32]. Fermenting RB using fermented food-derived, ornithine-producing bacterial strains would enhance the functionality of bioactive substances, including ornithine, as well as enhancing the savor and flavor of RB compared to raw RB, highlighting the need for relevant research. The bioactivity of microbial-fermented RB has been examined extensively, and studies have reported that fermented RB has antioxidant and whitening effects [21,33], boosts immunity [34], improves intestinal health [35,36], has anti-cancer effects [37], and prevents metabolic syndrome [38], muscle atrophy [39], atopic dermatitis [40], and ulcerative colitis [41]. Moreover, red yeast rice (RYR), a Chinese dietary seasoning prepared by fermenting rice with *Monascus purpureus*, has been used in Chinese medicine for centuries, and contains monacolins, which are a family of HMG-CoA reductase inhibitors [42,43]. Monacolin K is equivalent to the statin known as lovastatin. Statins are widely accepted drugs that lower blood lipids as an HMG-CoA reductase inhibitor [44]. However, taking statins induces side effects, such as muscle pain, since HMG-CoA reductase inhibitors reduce biologically essential cholesterol synthesis [44]. To prevent the side effects of statins, researchers have reported alternative and safe nutraceutical sources [42,43,45].

The intake of a high-fat and -cholesterol diet increases lipid peroxide content in tissues and serum; therefore, an excessive consumption of dietary fat or cholesterol induces oxidative damage in tissues. Thus, the ability of certain factors to reduce blood cholesterol content has been researched multilaterally to prevent cardiovascular diseases. Moreover, since obesity-preventive drugs and cholesterol reducers currently on the market have several adverse reactions, there is active ongoing research to explore natural bioactive substances and to develop functional foods.

Therefore, this study assessed the anti-obesity effects and improvement of lipid metabolism from consuming RB fermented with ornithine-producing *Weissella korneensis* DB1 isolated from kimchi in C57BL/6J mice with obesity and hyperlipidemia induced through a high-fat and -cholesterol diet, with a particular focus on investigating the effects of fermenting RB. 

## 2. Materials and Methods

### 2.1. Materials

Fresh RB milled within a week was purchased from CJ Cheiljedang (Suwon, Korea). All other reagents were guaranteed reagents.

### 2.2. Preparation of Fermented Rice Bran 

For fermentation, ornithine-producing *Weissells koreensis* DB1 (Mun and Chang, 2019) isolated from kimchi was inoculated onto a liquid deMan Rogosa and Sharpe (MSR; Difico, Sparks, MD, USA) medium and incubated for 24 h at 30 °C. The LAB incubated in an MSR (Difico) medium was centrifuged at 4 °C, 10,000× *g* for 20 min, and the retrieved bacteria were suspended in sterile distilled water to prepare LAB for RB fermentation. After adding 80 mL of triple distilled water to 20 g of RB, 1% (*w*/*v*) glucose and 1% arginine were added, and the solution was autoclaved at 121 °C. *W. koreensis* DB1 strain was inoculated into the autoclaved RB solution at 1.0 × 10^6^ cfu/g and incubated for 2 days at 30 °C. After fermentation, the fermented RB solution was hot air dried in a temperature and humidity chamber (HB-105SP, Hanbaek, Korea) for 12 h at 55 °C. The dried fermented RB was powdered using a grinder (BW-300, Boowon, Korea).

### 2.3. Animals and Experimental Design

Forty-six-week-old male C57BL/6J mice were purchased from Central Lab Animal Inc. (Seoul, Korea). The mice were fed a solid diet (Research Diets, New Brunswick, NJ, USA) for a week prior to the experiment to acclimate them to the environment, after which, they were divided into four groups of ten using a randomized block design and were reared for 10 weeks. Table 1 presents the experimental diet. The experimental groups were divided into the normal diet (ND) group, high-fat and -cholesterol diet (HFCD) group, high-fat and -cholesterol diet with 5% RB (HFCD-RB) group, and high-fat and -cholesterol diet with 5% fermented RB (HFCD-FRB) group. The HFCD consisted of 20% fat (41% of calories from fat) and 1.25% cholesterol based on the AIN-93G diet (D12451, Research Diets Inc., Brunswick, NJ, USA). Body weight was measured once a week during the rearing period, and food intake was measured at the same time every day by subtracting the amount left over from the amount served. Water and food were consumed ad libitum. The temperature and humidity were maintained at 18 ± 2 °C and 50–60%, respectively, with a 12 h light–dark cycle (08:00–20:00). 

### 2.4. Blood and Tissue Sample Processing

After the 10-week rearing period, the animals were sacrificed following a 12 h food restriction. The collected blood was refrigerated in a centrifuge tube for 1 h and centrifuged at 1100× *g*, 4 °C for 15 min. The serum was taken and stored in a deep freezer at –70 °C (VIPTM Series. SANYO Electrical Co., Ltd., Osaka, Japan) for the experiment. The liver and white fat pads (i.e., epididymal, mesenteric retroperitoneal and perirenal fat pads) were removed immediately after collecting the blood, washed with phosphate buffered saline, weighed, frozen with liquid nitrogen, and stored at –70 °C until analysis. Tissue weight was calculated as a relative weight against 100 g of food-restricted body weight before the autopsy. A part of the removed liver tissue was fixed in 4% paraformaldehyde solution for pathological analysis, and a part of the epididymal fat tissue was fixed in 10% formalin solution to measure the size of adipocytes. 

### 2.5. Serum, Hepatic, and Adipose Tissue Lipid Profiles

The serum triglyceride (TG), total cholesterol (TC) and HDL-cholesterol (HDL-C) contents were measured using an automatic biochemical analyzer (Fuji Dri-Chem 3500, Fujifilm., Tokyo, Japan). The non-HDL-C content was calculated by subtracting HDL-C from TC. TG/HDL-C and TC/HDL-C ratios were computed from the measured TG, TC, and HDL-C values. The total lipid (TL) content in the liver and fat tissues was measured after extracting the lipids via the Folch method [46], and the TG and TC contents were measured from a part of the extracted TL. The TG and TC contents were measured using the methods of Biggs et al. [47] and Zlatkis and Zak [48], respectively. 

### 2.6. Serum Biochemical Parameters

Serum alanine aminotransferase (ALT), aspartate aminotransferase (AST), alkaline phosphatase (ALP), and lactate dehydrogenase (LDH) activity and glucose content were analyzed using an automatic biochemical analyzer (Fuji Dri-Chem 3500, Fujifilm, Japan). The serum insulin content was measured using an insulin radioimmunoassay kit (EIKEN Chemical Co., Ltd., Tokyo, Japan), and serum leptin and adiponectin contents were analyzed using an ELISA kit (quantikine and immunoassay kit, R&D system, Minneapolis, MN, USA).

### 2.7. Histological Examination of the Liver

After fixing a part of the liver tissue in 4% paraformaldehyde solution, the sections were cut in 3–4-μm slices at –25 °C using a Cryocut Microtme (Leica CM1800. Wetzler, Germany) and attached to slides for drying. The slides were stained with Oil-Red O, washed, neutralized, dehydrated, and mounted for observation under an optical microscope (TS100, Nikon, Tokyo, Japan). 

### 2.8. Cell Size Measurement of Epididymal Fat Tissue

After cutting mice epididymal fat tissues into consistent sizes, they were fixed in 10% phosphate-buffered formalin solution. Excess fixative was removed with running water, and moisture in tissue was removed with ethanol. After removing alcohol in tissue with xylene, the tissues were embedded in paraffin and sectioned into 5 μm slices to prepare glass microscope slides. The slides were stained with hematoxylin and eosin dye, and the size of the fat cells for each group was computed using an image analyzer program (National Institute of Mental Health, Bethesda, MD, USA) under an optical microscope (TS100, Nikon, Tokyo, Japan). 

### 2.9. RNA Extraction and Real-Time Polymerase Chain Reaction (PCR) Analysis

Total mRNA was extracted using the trizol reagent (Ambion, Auatin, TX, USA), and cDNA was synthesized from the extracted RNA using a cDNA reverse transcription kit (Applied Biosystems, Foster city, CA, USA). Genes were expressed using the primer for each gene and SYBRGreen master mix (TOYOBO Co., Ltd. Osaka, Japan) and measured with the 7500 Real-Time PCR system (Applied Biosystems, Foster city, CA, USA). Table 2 presents the PCR primers used in this experiment.

### 2.10. Statistical Analysis

The experimental data were analyzed using a one-way analysis of variance (ANOVA; GraphPad PRISM 8, San Diego, CA, USA) and presented as the mean and standard error. Differences in the study parameters among the groups were analyzed with one-way ANOVA followed by Tukey’s post hoc test to verify significance at *p* < 0.05.

## 3. Results

### 3.1. Rice Bran or Fermented Rice Bran Ameliorated HFCD-Induced Weight Gain

**Figure 1:**
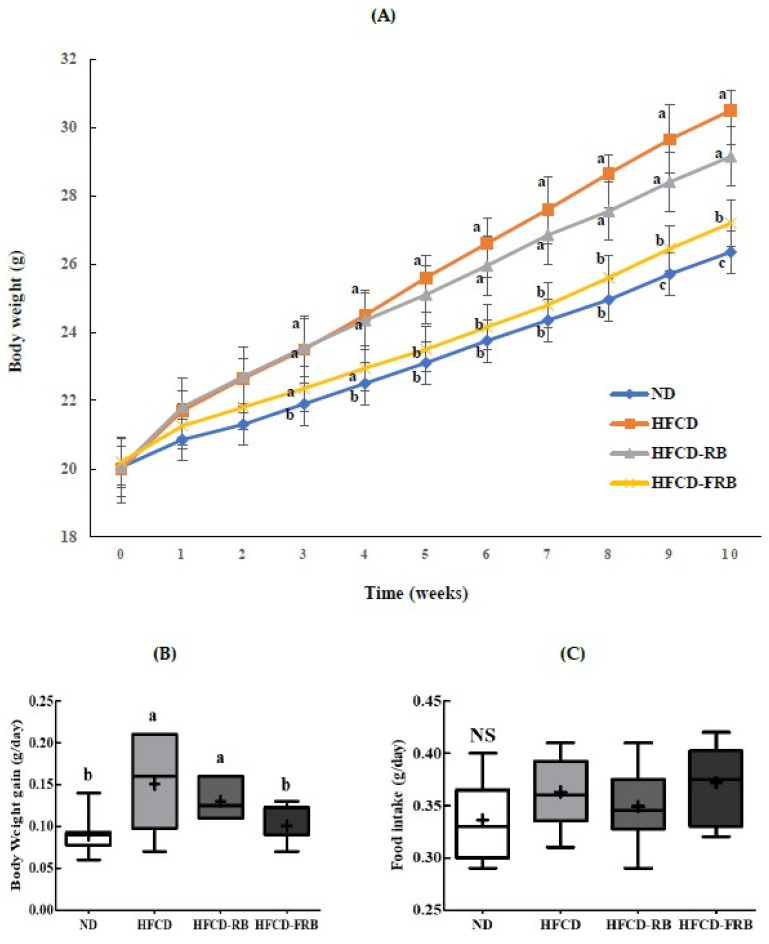
Body weight changes, daily body weight gain and daily average food intake in mice fed experimental diets for 10 weeks. Diet groups: ND, normal diet group; HFCD, high-fat and -cholesterol diet group; HFD-RB, HFCD + non-fermented rice bran powder group; HFCD-FCA, HFCD + fermented rice bran powder group. (**A**) Body weight was measured weekly, (**B**) daily body weight gain, and (**C**) daily average food intake were measured. Values are displayed as a Box-and-Whisker plot with means (expressed as ‘+’), *n* = 10. a–c: bars with different letters are significantly different at *p* < 0.05 by Tukey’s post hoc test. NS: Not significantly among the groups.

Figure 1 shows the changes in the body weight and food intake of mice during the 10-week experimental period. As shown in Figure 1A, all groups exhibited a body weight gain over the 10-week period, and the HFCD group had a significantly greater weight gain compared to the ND group. The amount of weight gain began to decrease from week three in the HFCD-FRB groups compared to the HFCD group, with the difference becoming significant from week five. The amount of daily weight gain in the HFCD-RB and HFCD-FRB groups decreased by 13.4% and 33.4%, respectively, compared to the HFCD group, and only the HFCD-FRB group showed a significant reduction compared to the HFCD group (Figure 1B). Food intake did not significantly differ among the experimental groups (Figure 1C). 

**Figure 2:**
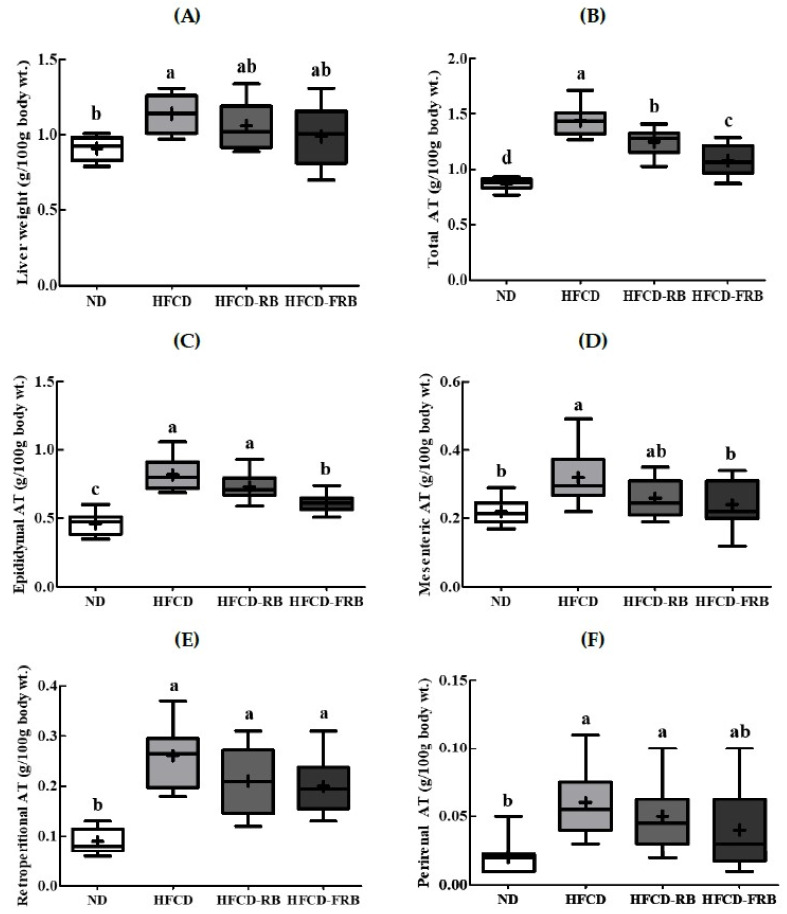
Relative hepatic tissue and white adipose tissue weight in mice fed experimental diets for 10 weeks. Diet groups: ND, normal diet group; HFCD, high-fat and -cholesterol diet group; HFD-RB, HFCD + non-fermented rice bran powder group; HFCD-FCA, HFCD + fermented rice bran powder group. Relative (**A**) hepatic tissue, (**B**) total white adipose tissue (total WAT), (**C**) epididymal adipose tissue (EAT), (**D**) mesenteric adipose tissue (MAT), (**E**) retroperitoneal adipose tissue (RAT), and (**F**) perirenal adipose tissue (PAT). Relative tissue weights (%) were calculated as organ weight (g)/final BW × 100. Values are displayed as a Box-and-Whisker plot with means (expressed as ‘+’), *n* = 10. a–d: bars with different letters are significantly different at *p* < 0.05 by Tukey’s post hoc test.

Figure 2 presents the relative weights of tissue, epididymal fat tissue, mesenteric fat tissue, retroperitoneal fat tissue, perirenal fat tissue, and total fat tissue per 100 g of body weight. The weight of the liver tissue was about 1.3 times greater in the HFCD group than in the ND group, and was about 7.1% and 13.2% lower in the HFCD-RB and HFCD-FRB groups, respectively, compared to the HFCD group (Figure 2A). There were no differences in the weight of the liver tissue between the FFCD-RB and HFCD-FRB groups. The total white fat pads were 1.7 times heavier in the HFCD group compared to the ND group, and 13.2% and 25.0% significantly lighter in the HFCD-RB and HFCD-FRB groups, respectively, compared to the HFCD group (Figure 2B). Compared to the ND group, the weights of visceral fat pads, namely epididymal fat tissue, mesenteric fat tissue, retroperitoneal fat tissue, and perirenal fat tissue, were about 1.8, 1.5, 2.9, and 1.3 times higher, respectively, in the HFCD group; however, they were 11.0–25.7%, 18.8–25.0% 7.7–15.4%, and 16.7–33.4% lower, respectively, in the HFCD-RB and HFCD-FRB groups compared to the HFCD group (Figure 2C–F). Compared to the HFC-RB group, the HFCD-FRB group had significantly lighter epididymal fat tissue, perirenal fat tissue, and total fat tissue weight. 

### 3.2. ALT, AST, ALP, and LDH Activities in Serum

**Figure 3:**
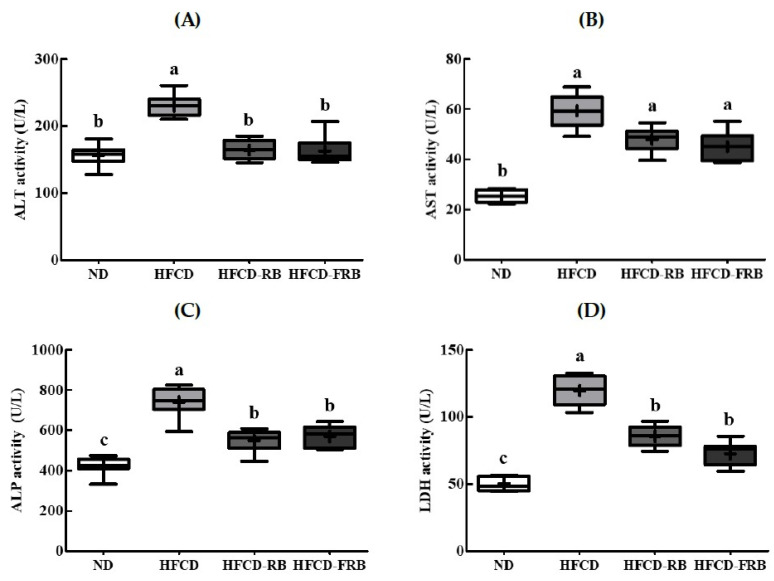
Biochemistry markers of hepatic function in mice fed experimental diets for 10 weeks. Diet groups: ND, normal diet group; HFCD, high-fat and -cholesterol diet group; HFD-RB, HFCD + non-fermented rice bran powder group; HFCD-FCA, HFCD + fermented rice bran powder group. (**A**) Alanine aminotransferase (ALT), (**B**) aspartate aminotransferase (AST), (**C**) alkaline phosphatase (ALP), and (**D**) lactate dehydrogenase (LDH) activities were analyzed biochemically. Values are displayed as a Box-and-Whisker plot with means (expressed as ‘+’), *n* = 10. a–c: bars with different letters are significantly different at *p* < 0.05 by Tukey’s post hoc test.

Figure 3 presents the results of ALT, AST, ALP, and LDH activities in mice fed HFCD with RB and fermented RB for 10 weeks. Serum ALT, AST, ALP, and LDH activities were 47.4%, 134.5%, 73.6%, and 138.6% higher in the HFCD group, respectively, compared to the ND group (Figure 3A–D). There were no significant differences in AST activity among HFCD groups (HFCD, HFCD-RB, HFCD-FRB). ALT, ALP, and LDH activities elevated through HFCD were significantly reduced by 28.6–29.3%, 25.8–23.1%, and 28.5–39.2%, respectively, through RB and fermented RB (HFCD-RB, HFCD-FRB). There were no differences in serum ALT, ALP, and LDH activities between the HFCD-FRB and HFCD-RB groups.

### 3.3. Lipid Profiles in Serum

**Figure 4:**
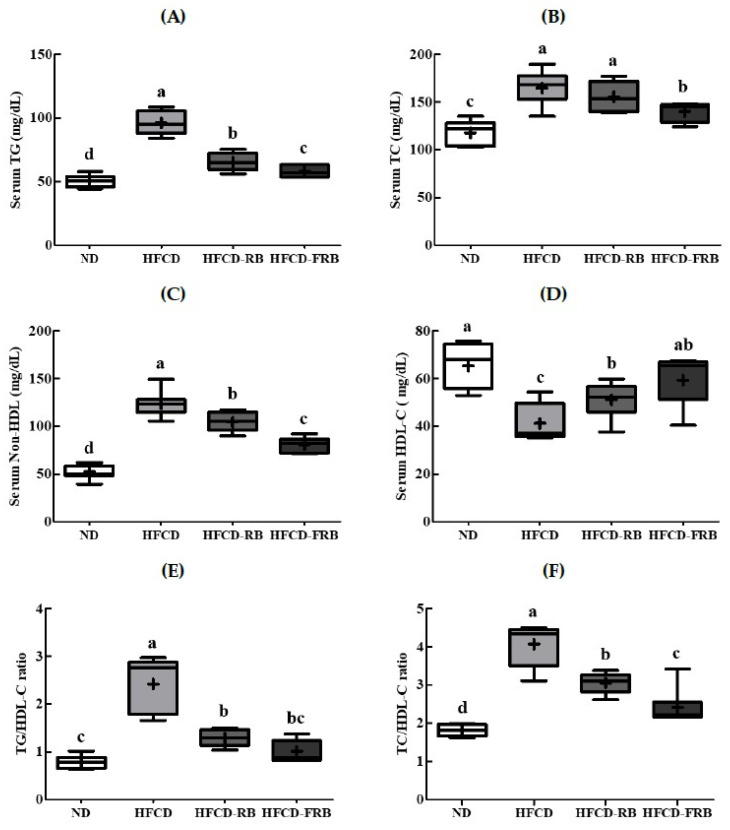
Lipid profiles, TG/HDL-C and TC/HDL-C ratios in mice fed experimental diets for 10 weeks. Diet groups: ND, normal diet group; HFCD, high-fat and -cholesterol diet group; HFD-RB, HFCD + non-fermented rice bran powder group; HFCD-FCA, HFCD + fermented rice bran powder group. Serum levels of (**A**) triglyceride (TG), (**B**) total cholesterol (TC), (**C**) non-high-density lipoprotein cholesterol (non-HDL-C), (**D**) high-density lipoprotein cholesterol (HDL-C), (**E**) TG/HDL-C and (**F**) TC/HDL-C ratios were measured in the experimental mice. Values are displayed as a Box-and-Whisker plot with means (expressed as ‘+’), *n* = 10. a–d: bars with different letters are significantly different at *p* < 0.05 by Tukey’s post hoc test.

Figure 4 presents the changes in lipid profiles in serum. Compared to the ND group, the HFCD group showed markedly increased serum TG (94.6%), TC (40.0%), and non-HDL-C contents (86.6%). However, compared to the HFCD group, the HFCD-RB and HFCD-FRB groups showed 32.2% and 39.5% lower serum TG content, 5.6% and 15.1% lower TC content, and 15.5% and 34.8% lower non-HDL-C content, respectively (Figure 4A–C). The HFCD group had 36.9% lower serum HDL-C content compared to the ND group, but the HFCD-RB and HFCD-FRB groups had 24.2% and 43.6% higher HDL-C content, respectively, compared to the HFCD group (Figure 4D). The TG/HDL-C and TC/HDL-C ratios were significantly higher in the HFCD group compared to the ND group, and were lower in the HFCD-RB and HFCD-FRB groups compared to the HFCD group (Figure 4E,F).

### 3.4. Leptin, Adiponectin, Glucose, and Insulin Contents in Serum

**Figure 5:**
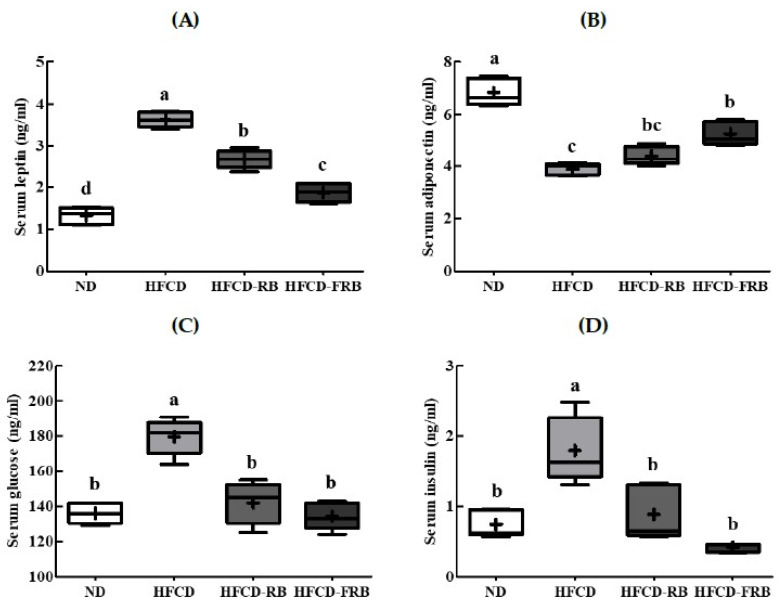
Leptin, adiponectin, fasting glucose, and insulin contents in mice fed experimental diets for 10 weeks. Diet groups: ND, normal diet group; HFCD, high-fat and -cholesterol diet group; HFD-RB, HFCD + non-fermented rice bran powder group; HFCD-FCA, HFCD + fermented rice bran powder group. Serum levels of (**A**) leptin, (**B**) adiponectin, (**C**) fasting glucose, and (**D**) insulin contents were measured in the experimental mice. Values are displayed as a Box-and-Whisker plot with means (expressed as ‘+’), *n* = 10. a–d: Bars with different letters are significantly different at *p* < 0.05 by Tukey’s post hoc test.

Figure 5 shows the changes in serum leptin, adiponectin, glucose, and insulin contents. The HFCD group had significantly higher serum leptin, glucose, and insulin contents and significantly lower adiponectin content compared to the ND group. The HFCD group had 2.7 times higher leptin content compared to the ND group, but the HFCD-RB and HFCD-FRB groups had 26.0% and 48.4% lower leptin content, respectively, compared to the HFCD group, with the HFCD-FRB group exhibited a level similar to that of the ND group (Figure 5A). The HFCD group had 0.6 times lower adiponectin content compared to the ND group, but the HFCD-RB and HFCD-FRB groups had 13.6% and 35.4% higher adiponectin, respectively, compared to the HFCD group (Figure 5B). The HFCD group had 2.4 times and 1.3 times higher insulin and glucose contents, respectively, compared to the ND group; however, the HFCD-RB and HFCD-FRB groups had 50.6–76.7% and 20.9–25.2% lower insulin and glucose contents, respectively, compared to the HFCD group (Figure 5C,D). There were no significant differences in serum adiponectin and insulin contents between the HFCD-RB and HFCD-FRB groups.

### 3.5. Hepatic Lipid Levels and Histopathological Changes 

**Figure 6:**
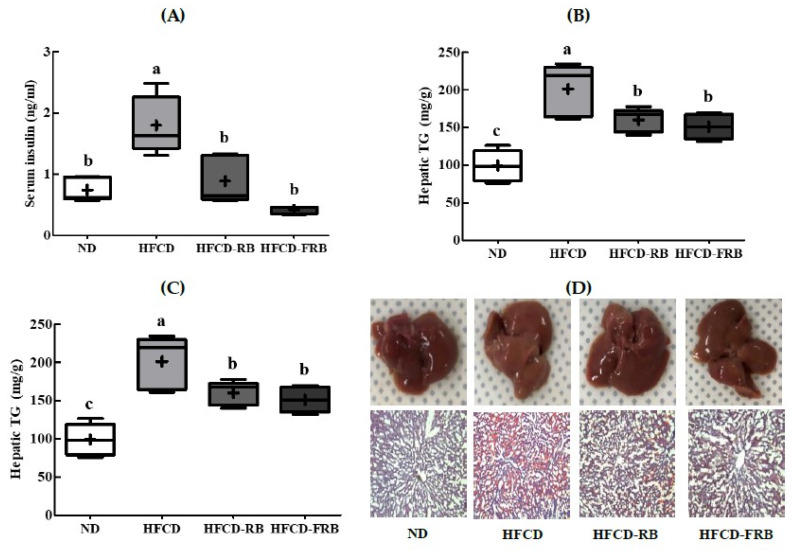
Hepatic lipid accumulation in mice fed experimental diets for 10 weeks. Diet groups: ND, normal diet group; HFCD, high-fat and -cholesterol diet group; HFD-RB, HFCD + non-fermented rice bran powder group; HFCD-FCA, HFCD + fermented rice bran powder group. (**A**) Hepatic lipid, (**B**) hepatic triglyceride (TG), and (**C**) hepatic total cholesterol were measured; (**D**) liver samples were sliced, fixed and stained with Oil-red O. Values are displayed as a Box-and-Whisker plot with means (expressed as ‘+’), *n* = 10. a–c: bars with different letters are significantly different at *p* < 0.05 by Tukey’s post hoc test.

Figure 6 shows the hepatic lipid levels and histopathological changes. The hepatic TL, TG, and TC contents were significantly higher in the HFCD group than in the ND group. The hepatic TL content was 19.2% and 46.5% lower in the HFCD-RB and HFCD-FRB groups, respectively, compared to the HFCD group; however, the HFCD-FRB group exhibited a level similar to that of the ND group (Figure 6A). The changes in hepatic TG and TC contents were similar to that of the TL content, where the HFCD-RB and HFCD-FRB groups showed lower levels compared to the HFCD group (Figure 6B,C). In particular, the HFCD-FRB group had significantly lower hepatic TL and TC contents compared to the HFCD-RB group. Figure 6D shows the results of fatty deposition in liver tissues observed with Oil-Red O staining under an optical microscope. Compared to the ND group, there were clearly more fatty deposits stained in red in the liver tissues of the HFCD group. While both the HFCD-RB and HFCD-FRB groups showed fatty deposition in the liver, these groups had markedly lower areas stained in red compared to the HFCD group.

### 3.6. Expression of mRNA of Enzyme-Related to Fat Metabolism in Liver 

**Figure 7:**
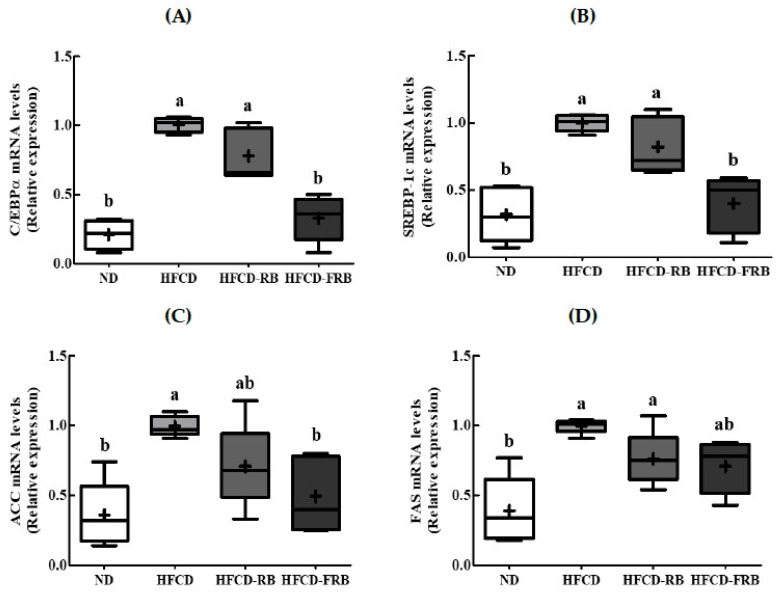
Hepatic mRNA expression in mice fed experimental diets for 10 weeks. Diet groupsL ND, normal diet group; HFCD, high-fat and -cholesterol diet group; HFD-RB, HFCD + non-fermented rice bran powder group; HFCD-FCA, HFCD + fermented rice bran powder group. (**A**) CCAT-enhancer-binding protein-alpha (C/EBPα), (**B**) sterol regulatory element binding protein-1c (SREBP-1c), (**C**) acetyl-CoA carboxylase (***ACC***), and (**D**) fatty acid synthase (FAS) mRNA expression was analyzed by qRT-PCR and normalized by β-actin. Values are displayed as a Box-and-Whisker plot with means (expressed as ‘+’), *n* = 10. a,b: bars with different letters are significantly different at *p* < 0.05 by Tukey’s post hoc test.

To examine the mechanism underlying the changes in the serum and hepatic lipid profiles with RB and fermented RB intake, we analyzed the effects of lipid metabolism-related enzymes in the liver on gene expression (Figure 7). The expression of lipogenic transcription factors CCAT-enhancer-binding protein-alpha (C/EBPα) and sterol regulatory element binding protein-1c (SREBP-1c) was significantly upregulated in the HFCD group than in the ND group, while it was significantly reduced in the HFCD-RB and HFCD-FRB groups compared to the HFCD group (Figure 7A,B). The mRNA expression for ACC and FAS, major enzymes related to lipogenesis in the liver, was lower in the HFCD-RB and HFCD-FRB groups compared to the HFCD group (Figure 7C,D). Only the HFCD-FRB group had a significantly lower FAS gene expression compared to the HFCD group. 

### 3.7. Epididymal Adipose Tissue Lipid Levels and Histopathological Changes 

**Figure 8:**
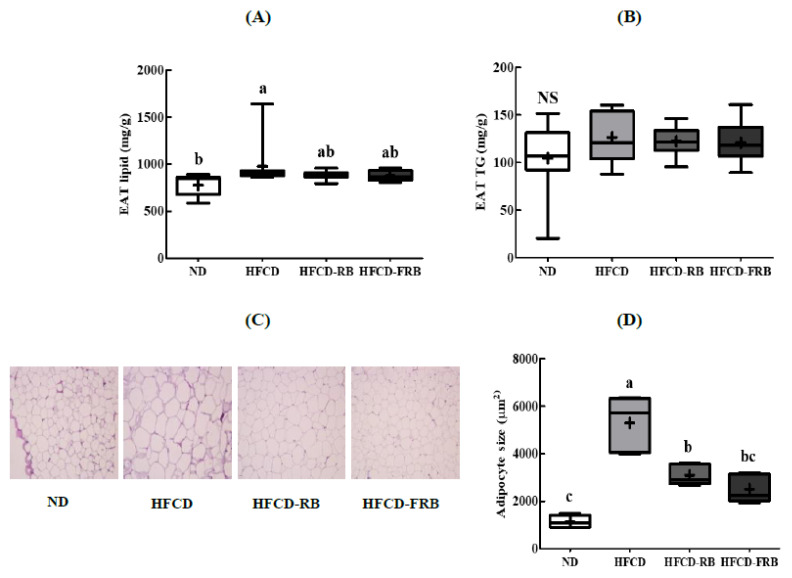
Epididymal adipose tissue lipid profiles and adipocyte size in mice fed experimental diets for 10 weeks. Diet groups: ND, normal diet group; HFCD, high-fat and -cholesterol diet group; HFD-RB, HFCD + non-fermented rice bran powder group; HFCD-FCA, HFCD + fermented rice bran powder group. (**A**) Lipid, and (**B**) triglyceride (TG) were measured from the epidydimal adipose tissue (EAT); (**C**,**D**) EAT was stained with hematoxylin and eosin (HE). Magnification, 100×. Values are displayed as a Box-and-Whisker plot with means (expressed as ‘+’), *n* = 10. a–c: bars with different letters are significantly different at *p* < 0.05 by Tukey’s post hoc test.

Figure 8 shows the lipid content and size of fat cells in epididymal fat pads. The HFCD group had significantly greater TL and TG contents in epididymal fat tissues. The HFCD-RB and HFCD-FRB groups had significantly lower TL and TG contents in the epididymal fat tissues compared to the HFCD group (Figure 8A,B). The HFCD group had markedly larger fat cells compared to the ND group, while the HFCD-RB and HFCD-FRB groups had smaller fat cells compared to the HFCD group (Figure 8C,D). There were no differences between the HFCD-RB and HFCD-FRB groups.

## 4. Discussion

Recently, the obesity population tends to have a lower carbohydrate diet to overcome extra fat gain from the carbohydrate sources. However, extremely limited carbohydrate consumption may generate excess ketone bodies and/or muscle loss. Therefore, the metabolic complicated population may take enough carbohydrate sources as a diet. If the carbohydrate source has functions to combat with metabolic complications, then it may be easily achievable and effective. RB is a carbohydrate source, and it has anti-obesity and anti–dyslipidemic functions. Dietary fiber accounts for about 25% of RB, and dietary fibers in RB consist of cellulose, hemicellulose, lignin, pectin, and gum [49]. However, the cell wall of RB predominantly comprises hemicellulose and thus is not digested in the body, which hinders the absorption of the functional ingredients of RB. In addition, although RB contains about 1–2% phenolics, and 70% of these phenolic compounds exist as esters in the arabinoxylan residues in the cell wall [50], it is difficult to extract ester-bound phenolics with organic solvents or water [51]. Fermentation occurs by adding sugar, enzymes, or microbes such as LAB in a raw material. This process activates various enzymes in the raw material and produces several functional ingredients, converting the bioactive ingredients in the raw material to easily digested or absorbed forms [52]. Thus, many studies are underway to investigate the LAB fermentation of natural food materials and to develop functional food products with health benefits. LAB-fermented RB is known to produce amino acids as well as biologically active metabolites, such as phenolic acid, ferulic acid, vanillic acid, protocatechuic acid, γ-oryzanol, phytic acid, inositol, β-sitosterol, acetic acid, vitamin E, vitamin B, lipids, and proteins [21,22]. Active ingredients in yeast-fermented red rice (RYR) also include a number of monacolins, most importantly monacolin K, pigments, organic acids, amino acids, sterols, decalin derivates, flavonoids, lignans, coumarin, terpenoids, and polysaccharides [53]. Monacolin K is known as lovastatin, the active ingredient in the prescription drug Mevacor. Moreover, there are unknown functional substances that may be generated during fermentation by yeasts and microorganisms. Thus, fermented RB or RYR is believed to be beneficial for patients with obesity or patients with hyperlipidemia due to its nutrients and bioactive substances [21,22,30,33,34,53]. 

Kimchi contains microorganisms of various genera, including *Lactobacillus, Leuconostoc, Pediococcus,* and *Weissella*, and these are known to play a crucial role in the fermentation of kimchi [27]. Particularly, RB fermented with ornithine-producing *W. koreenisis* DB1 isolated from kimchi was found to contain high amounts of organic acids that regulate lipid metabolism and have anti-obesity effects that are not found in raw RB [54]. Fermented RB also had greater ornithine and citrulline contents compared to non-fermented RB [29].

Therefore, this study was conducted to develop processed RB products and examine the activities in these products. To this end, we investigated obesity prevention and improvement of lipid metabolism in mice with obesity and hyperlipidemia induced by a high-fat (45% of calories), high-cholesterol (1.25% of diet) diet after the consumption of RB powder fermented using ornithine-producing *W. korenisis* DB1 isolated from kimchi. Six-week-old C57BL/6 mice that were fed an HFCD for 10 weeks were significantly heavier, had a greater weight of fat tissues, including visceral fat, and had larger fat tissues compared to mice that were fed a normal diet. The elevated levels of serum TG and TC and hepatic TC confirm that non-alcoholic fatty liver was induced, and histopathological examination of liver tissue confirmed that the adipocyte count and hepatic steatosis in the liver had increased. The increased expression of lipogenic transcription factors C/EBPα and SREBP-1c and increased mRNA expression of FSA and ACC, which are key enzymes related to lipogenesis, confirmed that fat accumulation and dyslipidemia were induced.

Regarding changes in body weight, the groups given non-fermented RB and fermented RB showed slower weight gain compared to the HFCD group. While the RB groups did not differ in the rate of weight gain, the fermented RB group exhibited a better suppression of weight gain. Alauddin et al. [40] also reported that the suppression of weight gain was greater in mice given fermented RB than in those given raw RB. Our results correspond to past findings that providing study animals a high-fat diet with RB or stabilized RB leads to lower weight gain compared to the control group [18,55,56] and that only a high-cholesterol diet with RB suppresses weight gain, with other grain brans being unable to suppress weight gain [57]. Adding bran unsaponifiable matter or rice bran polysaccharide to a high-fat diet also affects weight change [58,59]. Various bioactive ingredients in regular RB or fermented RB that are involved in weight regulation, such as dietary fiber, polysaccharide, phenolic compounds, amino acid, lipid, organic acids, and protein, are presumed to affect weight changes in obesity-induced mice. 

Visceral fat, including epididymal fat, is a major site of inflammation. Increased inflammatory markers as a result of excessive fat buildup and fat tissue dysfunction induce insulin resistance, which in turn causes impaired glucose tolerance and hyperinsulinemia, and is associated with diabetes mellitus and cardiovascular diseases [60,61,62]. In this experiment, the relative weight of major fat tissues was calculated by multiplying the weight (g) of each organ by 100 and dividing it by the weight of the mouse. The consumption of RB or fermented RB powder is anticipated to lower the risk of chronic disease by reducing the buildup of visceral fat. RB or fermented RB intake reduced epididymal TL and TC contents and the size of fat cells, indicating that it suppressed fat buildup. Organic acid acetic acid is known to regulate lipid metabolism in the body [63]. The fermented RB used in this study contained 25.77 g/kg of acetic acid not found in non-fermented RB [30]. Compared to non-fermented RB, RB fermented with *Pediococcus acidilactici* has an abundant amount of acetic acid, lactic acid, and ferulic acid and has two-fold higher γ-oryzanol and α-tocopherol [22]. After the oral administration of RB polysaccharide with a high-fat diet for 10 weeks, these mice had lighter fat tissue compared to mice that were only fed a high-fat diet [59]. Further, treating 3T3-L1 cells with *W. koreenisis* OK1-6, an ornithine-producing strain isolated from kimchi, has been reported to suppress fat accumulation [64]. In our study, fermented RB had superior effects to RB in suppressing body fat buildup, and this seems to be due to the ingredients known to be involved in fat buildup, namely acetic acid, ornithine, and citrulline, produced during the fermentation process by *W. koreensis* DB1, a strain isolated from kimchi. 

Elevated activities of ALT, AST, ALP, and LDH in the blood in response to the HFCD are used as indices of liver damage [65]. Healthy people have low concentrations of these enzymes in the blood and a relatively high concentration in liver cells. Obesity, which is a metabolic disorder, also increases the concentration of these enzymes due to abnormal liver metabolism, as carbohydrates and fatty acids are not used as an energy source [66]. Furthermore, in people with liver damage, such as those with liver diseases or those who have had hepatectomy, these enzymes are released from liver cells into the blood and thus are present in high concentrations in the blood [65]. In this study, elevated AST activity due to the HFCD tended to decrease—although insignificantly—with RB or fermented RB intake, and serum ALT, ALP, and LDH activities significantly decreased with RB or fermented RB intake. Thus, RB or fermented RB powder is anticipated to have positive effects in maintaining serum and hepatic functioning. Kim et al. [36] also reported that hot water extract of RB fermented with *Saccharomyces cerevisiae* IFO 2346 reduces GPT and LDH activity in the blood.

Excessive animal fat intake and the consequent high cholesterol and high triglyceride concentration in the blood are considered an important cause of metabolic syndrome [67]. Serum TG concentration is elevated with high calorie or fat intake, and hypertriglyceridemia is known to be a cause of diabetes mellitus, hypertension, and cardiovascular diseases. In addition to blood TG concentration, TC concentration also serves as an important marker for determining hyperlipidemia [68]. In this study, serum TG, TC, and non-HDL-C concentrations decreased with RB and fermented RB intake. The TG/HDL-C and TC/HDL-C ratios are used to predict myocardial infarction and coronary heart disease, respectively, and people with metabolic syndrome are known to have dyslipidemia with high TG, LDL-C, TG/HDL-C ratio, and TC/HDL-C ratio [69]. The TG/HDL-C and TC/HDL-C ratios also markedly increased with the HFCD but were reduced with RB and fermented RB intake. Similarly, adding RB enzymatic extract to a high-fat diet or an HFCD leads to lower TG and TC levels [70,71]. Orally administering RB water extract with a high-fat diet for four weeks in mice was reported to improve serum lipid metabolism and exhibited vasoprotective effects [56], and the phenolic compounds in the RB were considered to be involved in these effects [6,7]. Polyphenol compounds are known to be beneficial for cardiovascular diseases by reducing blood lipid concentration [69,72,73], and ferulic acid, a phenolic compound that is abundant in RB, also improved blood lipid metabolism [74]. Therefore, the continued consumption of RB and fermented RB containing diverse bioactive compounds would lower the risk for metabolic syndrome by increasing serum HDL-C and reducing TG and TC concentrations as well as TG/HDL-C and TC/HDL-C ratios. 

The HFCD group had significantly higher hepatic tissue weight relative to body weight and TG and TC concentrations compared to the ND group. This is similar to previous reports that a high-fat diet causes fat buildup in the liver and increases the weight of the liver [75], and that adding a high-cholesterol diet further increases the weight of the liver by causing TC and TG buildup in the liver tissues [76]. A high-fat diet is reported to cause liver steatosis and enlarged liver, and thus increases the weight of the liver, as it causes a buildup of cholesterol in liver tissues by inhibiting normal excretion of cholesterol [77]. In this study, RB and fermented RB powder were found to suppress fat buildup, as evidenced by the reduced weight of liver tissue and lipid levels in the liver. Considering the report that phenolic compounds in natural plants have anti-obesity effects, as they form strong compounds with enzymes or proteins and thus activate pancreatic phospholipase and hinder lipid absorption in the intestine [78], phenolic compounds in RB and fermented RB powder are speculated to contribute to reducing hepatic lipid content. One study found that RB phenolic compounds protect against liver steatosis by inhibiting oxidative stress in mice fed a high-fat diet [6]. Further, RB phenolic compounds were found to inhibit ethanol-induced liver damage by improving lipid metabolism in mice liver [7].

Changes in blood and hepatic lipid contents are regulated by various mechanisms, including hepatic lipid synthesis, degradation, and oxidation. ACC facilitates lipogenesis by converting acetyl-CoA to malonyl-CoA, and the latter inhibits fat oxidation by inactivating the expression of CPT-1 [79]. FAS is an important enzyme that produces fatty acids from acetyl-CoA and malonyl-CoA, and it is expressed as carbohydrate intake that induces the transcription of its gene [80]. SREBP-1c expression is induced early in the differentiation of fat cells, and it facilitates the activity of PPARγ, thereby upregulating the expression of acyl-CoA synthase, which promotes a buildup of triglycerides [81]. When RB is orally administered to mice fed a high-fat diet, the phenolic compounds in the RB regulate lipid metabolism by activating 5‘-adenosine phosphate-activated protein kinase α and suppressing the expression of the SREBP-1c gene [6]. In our study, hepatic ACC and FAS expression were the lowest in the HFCD-FRB group, which suggests that fermented RB suppresses lipogenesis more than raw RB. In addition, the increased levels of ornithine or various functional ingredients as a result of fermentation seem to have influenced the regulation of the expression of hepatic lipogenic genes SREBP-1c and C/EBPα, an HFCD-induced obesity mice model, thereby leading to reduced body weight and body fat. Kimchi fermented with ornithine-producing *W. koreenisis* OK1-6 was found to have anti-obesity effects in C57BL/6J mice with high-fat diet-induced obesity by downregulating adipogenic and lipogenic gene expression [82]. In addition, acetic acid is known to reduce hepatic fat oxidation and body fat by increasing energy consumption, and is also reported to suppress the expression of lipogenic genes such as ACC, SREBP-1c, and FAS, to contribute to lowered body fat [83]. These results suggest that RB and fermented RB seem to have anti-obesity effects by regulating the expression of hepatic lipogenic genes and thus reducing body weight and body fat and affecting blood lipid metabolism. 

It is well known that fat tissues function in the synthesis and storage of triglycerides and as an endocrine organ, secreting adipokines that are involved in various signaling pathways in the body, including adiponectin, leptin, resistin, tumor necrosis factor alpha, and interleukin-6 [84]. Of them, leptin inhibits food intake by signaling satiety to the hypothalamus and facilitates energy consumption by activating the sympathetic nervous system. Adiponectin induces weight loss by enhancing insulin sensitivity in fat, muscle, and liver tissues, maintaining glucose homeostasis, increasing fat oxidation, and inhibiting lipid accumulation [85]. Adding RB or fermented RB, which is a rich source of fibers and phytochemicals, to an HFCD reduced serum leptin levels while increasing adiponectin levels, suggesting that it reduces body fat. Further, it is anticipated to improve metabolic disorders by improving fasting glucose and insulin concentrations. Providing 5% RB or fermented RB to stroke-prone spontaneously hypertensive rats also led to similar results, and fermented RB was superior to raw RB in regulating adipokines secretion and reducing blood glucose [40]. Feeding obese mice water-soluble RB enzymatic extract for 20 weeks enhanced insulin resistance and increased serum adiponectin concentration [70]. RB water extract was reported to prevent metabolic syndrome by increasing insulin resistance and inhibiting abdominal fat accumulation when orally administered to mice for four weeks with a high-fat diet [56]. Stabilized RB has been reported to reduce blood glucose in rats and humans [11,18]. Further, feeding diabetic mice RB-extracted γ-oryzanol reduced fasting glucose and insulin concentration [86], and consuming β-sistosterol in RB was also found to reduce blood glucose [87]. The regulation of adipokine secretion and reduction in blood glucose with the consumption of RB are speculated to be influenced by a synergic interaction among fibers and various other effective ingredients of RB such as γ-oryzanol, β-sistosterol, and tocotrienol.

Feeding mice fermented RB with an HFCD reduced body weight and fat tissue, reduced blood lipid concentration, reduced the number and size of adipocytes in hepatic tissues, reduced the size of epididymal fat cells, and downregulated lipogenic gene expression, which prevented fat accumulation, thereby preventing obesity and improving lipid metabolism. This seems to be attributable to polyphenol compounds, flavonoid, acetic acid, GABA, γ-oryzanol, citrulline, and ornithine in fermented RB. LAB-fermented RB contains high lactic acid and acetic acid content and γ-oryzanol, α-tocopherol, and ferulic acid levels, compared to raw RB [22,30,88]. In particular, the fermented RB used in this study was fermented using an ornithine-producing strain isolated from kimchi, and it contained a markedly higher level of ornithine and citrulline [29]. L-ornithine is widely used as a food material for increasing muscle synthesis by facilitating the secretion of growth hormones and preventing obesity by increasing basal metabolism [89], and L-citrulline is also reported to have anti-obesity effects [90].

## 5. Conclusions

In conclusion, fermented RB seems to prevent body fat synthesis and accumulation as well as hyperlipidemia by improving lipid metabolism. This suggests that fermented RB has the potential as a functional ingredient to improve obesity and dyslipidemia and reduce fasting glucose. In addition, the results suggest that antioxidant vitamins and minerals and other bioactive substances such as polyphenol, flavonoid, acetic acid, ornithine, and citrulline in fermented RB improve lipid metabolism and prevent cardiovascular diseases by suppressing lipogenesis and alleviating oxidative stress in liver tissues. Thus, fermented RB, which contained a markedly higher level of these ingredients, was superior to raw RB in its anti-obesity effects and its improvement of lipid metabolism. From an industrial perspective, the *W. koreensis* DB1 strain can potentially be used to increase the ornithine content in foods such as RB. Fermentation enhances the effect of RB in preventing metabolic syndrome, and RB powder is anticipated to be useful as a functional food product.

## Figures and Tables

**Table 1:** Composition of experimental diet.

(g/kg)					
	Groups	ND ^(1)^	HFCD ^(2)^	HFCD + RB ^(3)^	HFCD + FRB ^(4)^
Ingredients					
Casein		200.000	200.000	193.000	188.600
L-cystine		3.000	3.000	3.000	3.000
Corn starch		397.486	254.966	228.266	232.966
Maltodextrose		132.000	132.000	132.000	132.000
Sucrose		100.000	100.000	100.000	100.000
Cellulose		50.000	50.000	43.800	42.800
Lard			130.000	130.000	130.000
Soybean oil		70.000	70.000	59.900	60.600
Cholesterol			12.500	12.500	12.500
Mineral mix ^(5)^		35.000	35.000	35.000	35.000
Vitamin mix ^(6)^		10.000	10.000	10.000	10.000
Choline chloride		2.500	2.500	2.500	2.500
*tert*-Butylhydroquinone		0.014	0.034	0.034	0.034
Rice bran				50.000	
Fermented rice bran					50.000
Total		1000.0	1000.0	1000.0	1000.0
Total energy (kcal)		3960.0	4636.4	4657.7	4667.1
Fat (kcal %)		15.9	41.2	41.1	41.0

^(1)^ ND: normal diet. ^(2)^ HFCD: high-fat and high-cholesterol diet. ^(3)^ HFCD-RB: HFCD-rice bran. ^(4)^ HFCD-FRB: HFCD-fermented rice bran. ^(5)^ AIN-93-GX mineral mixture and ^(6)^AIN-93-VX vitamin mixture.

**Table 2:** Real-Time Polymerase Chain Reaction (RT-PCR) primer sequences for quantitative real-time (5′ to 3′).

Transcript	Forward Primer	Reverse Primer
C/EBPα	GTGTGCACGTCTATGCTAAACCA	GCCGTTAGTGAAG AGTCTCAGGTTT
SREBP-1c	GATCAAAGAGGAGCCAGTGC	TAGATGGTGGCTGCTGAGTG
ACC	CAACGCCTTCACACCACCTT	AGCCCATTACTTCATCAAAGATCCT
FAS	GGAACTGAACGGCATTACTCG	CATGCCGTTATCAACTTGTCC
β-actin	GTGGGGCGCCCCAGGCACCAGGGC	CTCCTTAATGTCACGCACGATTTC

C/EBPα, CCAAT-enhancer-binding protein α: SREBP-1c, sterol regulatory element-binding protein-1c: ACC, acetyl CoA carboxylase: FAS, fatty acid synthase.

## Data Availability

The data presented in this study are available from the corresponding author upon request.
